# Human Adenovirus Associated with Severe Respiratory Infection, Oregon, USA, 2013–2014

**DOI:** 10.3201/eid2206.151898

**Published:** 2016-06

**Authors:** Magdalena Kendall Scott, Christina Chommanard, Xiaoyan Lu, Dianna Appelgate, LaDonna Grenz, Eileen Schneider, Susan I. Gerber, Dean D. Erdman, Ann Thomas

**Affiliations:** Oregon Public Health Division, Portland, Oregon, USA (M. Kendall Scott, A. Thomas);; Centers for Disease Control and Prevention, Atlanta, Georgia, USA (C. Chommanard, X. Lu, E. Schneider, S.I. Gerber, D.D. Erdman);; PeaceHealth Department of Quality and Improvement, Springfield, Oregon, USA (D. Appelgate);; Oregon Public Health Laboratory, Hillsboro, Oregon, USA (L. Grenz)

**Keywords:** Human adenovirus, Human adenovirus 7, respiratory infection, outbreak, pneumonia, Oregon, viruses

## Abstract

Several human adenoviruses (HAdVs) can cause respiratory infections, some severe. HAdV-B7, which can cause severe respiratory disease, has not been recently reported in the United States but is reemerging in Asia. During October 2013–July 2014, Oregon health authorities identified 198 persons with respiratory symptoms and an HAdV-positive respiratory tract specimen. Among 136 (69%) hospitalized persons, 31% were admitted to the intensive care unit and 18% required mechanical ventilation; 5 patients died. Molecular typing of 109 specimens showed that most (59%) were HAdV-B7, followed by HAdVs-C1, -C2, -C5 (26%); HAdVs-B3, -B21 (15%); and HAdV-E4 (1%). Molecular analysis of 7 HAdV-B7 isolates identified the virus as genome type d, a strain previously identified only among strains circulating in Asia. Patients with HAdV-B7 were significantly more likely than those without HAdV-B7 to be adults and to have longer hospital stays. HAdV-B7 might be reemerging in the United States, and clinicians should consider HAdV in persons with severe respiratory infection.

## Abstract 2

HAdV-B7 might be reemerging in the United States.

Human adenoviruses (HAdVs) are a common cause of respiratory infection in persons of all ages. Acute upper and lower respiratory tract diseases, including pneumonia and bronchitis, have been attributed to HAdVs. Although many infections are mild, some persons, such as very young children, elderly or immunocompromised persons, or persons who have underlying pulmonary or cardiac disease, might be at increased risk for severe disease (*1*–*4*). HAdV infection can occur sporadically, endemically, or epidemically and often is influenced by HAdV species and type (*4*). Common settings for infection include the community, military recruit training centers, hospitals, and chronic care facilities (*3*,*5*–*8*).

HAdV comprises 7 species (A–G), and >51 types have been characterized by immunotypic and molecular methods. HAdVs that are most often associated with symptomatic respiratory infections include species B (types 3, 7, 14, and 21), species C (types 1, 2, and 5), and species E (type 4) (*9*). Infections with HAdV-C often are endemic, mild, and most commonly seen in young children (*2*). HAdVs can be shed from the respiratory and gastrointestinal tracts for weeks or longer, even in persons who are no longer symptomatic, especially young children and immunocompromised persons (*10*). In comparison, HAdV-B– and HAdV-E–associated respiratory infections are more commonly seen as part of an epidemic or as sporadic cases in adults, and infections with these viruses are often more severe (*11*).

Circulating HAdVs can vary temporally and geographically; emergent genomic variants are possibly associated with more severe illness (*8**,**12**,**13*). Recent reports have noted severe respiratory disease associated with the reemergence of HAdV-B7 and genomic variant 7d in China and other countries in Asia (*14*–*16*). However, HAdV-B7 was rarely reported in the United States during the past decade (*17*,*18*). Among a convenience sample of 291 specimens sent to the Centers for Disease Control and Prevention (CDC) from mid-2004 through 2013 for HAdV typing, only 7 (2.4%) were identified as HAdV-B7 (D.D. Erdman, pers. comm.).

In March 2014, clinicians in the metropolitan areas of Eugene and Portland, Oregon, USA, reported an increase in the number of HAdV detections among specimens from hospitalized patients with severe respiratory infections to the Oregon Public Health Division (OPHD). In this study, we describe the clinical, epidemiologic, and viral molecular features of this cluster of HAdV-positive cases identified during October 2013–July 2014.

## Methods

OPHD asked 3 major hospital systems that perform HAdV diagnostic testing to participate in the investigation. The hospital systems comprised 14 facilities located primarily within the Eugene and Portland metropolitan areas. Facilities averaged 210 beds (range 25–688) and 11,774 admissions annually (range 1,263–35,614). These hospital systems were asked to provide information on HAdV-positive specimens from patients with respiratory infections diagnosed after September 2013. Requested information comprised basic demographic and clinical data and whether specimens were available for HAdV typing. On April 19, 2014, OPHD released a statewide notice through the Health Alert Network requesting providers in Oregon to consider HAdV in the differential diagnosis for patients presenting with severe pneumonia or for unusual clusters of pneumonia.

We also obtained historical HAdV detection data from Oregon clinical laboratories that reported to the National Respiratory and Enteric Virus Surveillance System to review recent trends in HAdV detections. In addition, 2 large Oregon hospital systems with available HAdV detection data compared HAdV detections for November 2013–April 2014 with those for November–April from the respiratory disease seasons of the previous 3 years (i.e., 2010–11, 2011–12, 2012–13).

The study population comprised persons in whom HAdV was detected in respiratory specimens during October 2013–July 2014 by hospital laboratories that perform virus isolation, direct fluorescent antibody, or PCR for HAdV detection (retrospective and prospective) and were willing to submit data to OPHD and CDC. A case-patient was defined as a person with respiratory symptoms and a positive HAdV laboratory test result. Illness was defined as severe if the patient was hospitalized.

We reviewed available medical records and collected the following information: basic demographic information, symptom onset date, symptoms, hospital admission and discharge dates, intensive care unit (ICU) admission, use of mechanical ventilation, specimen type and collection date, and HAdV laboratory results. The medical record review was conducted under Oregon’s special study statute for issues of public health significance (http://arcweb.sos.state.or.us/pages/rules/oars_300/oar_333/333_019.html).

Available clinical specimens were shipped to the Oregon State Public Health Laboratory and then submitted to the CDC for HAdV confirmation and molecular typing. HAdV-positive specimens were typed by either conventional PCR and sequencing of HAdV hexon gene hypervariable regions 1–6 or by HAdV type-specific real-time PCR assays, as previously described (*19*,*20*). For enhanced genetic comparisons, genomic sequencing was performed on 7 HAdV-B7–positive samples collected during January 2014–May 2014. Deep sequencing libraries were prepared by using the Nextera XT DNA Sample Prep Kit and sequenced (250-bp paired-end sequencing) on an Illumina MiSeq Desktop Sequencer (both from Illumina, San Diego, CA, USA) (protocol available on request). In silico genome restriction enzyme digestion profiles were generated by using NEBcutter V2.0 (*21*), and genome-type determinations were based on the classification system previously described (*22*). Sequences were compared with HAdV-B7 genome type d (HAdV-B7d) reference strains 0901HZ/Shx/CHN/2009 (GenBank accession no. JF800905.1) and human/CHN/DG01/2011/7[P7H7F7] (GenBank accession no. KC440171.1). We used sequence alignment and neighbor-joining phylogenetic tree construction to compare the phylogenetic relationships among a representative sample of HAdV-B7 genomic sequences using ClustalW implemented in BioEdit version 7.0.5 (*23*) and MEGA7, respectively (*24*). We excluded cases determined by CDC to be HAdV-negative.

We used Excel (Microsoft Corp., Redmond, WA, USA) for data entry and SAS version 9.3 (SAS Institute, Cary, NC, USA) for data analysis. Mantel-Haenszel χ^2^ was used to assess associations. A p value <0.05 was considered significant.

## Results

**Figure 1:**
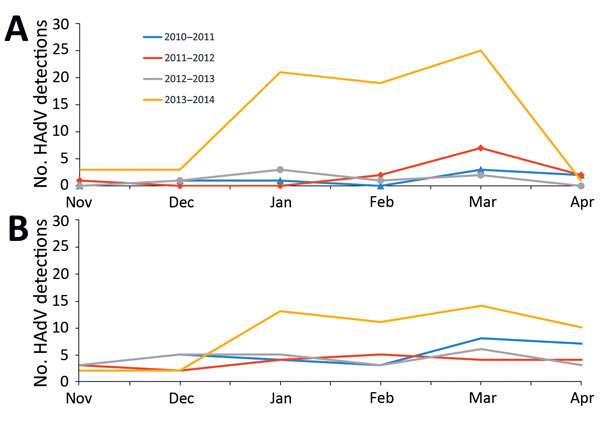
HAdV detections from 2 major hospital systems (A and B), Oregon, USA, November–April 2010–2014. Historical data collected by the Oregon Public Health Division. Data for hospital system C were not available. HAdV, human adenovirus.

**Figure 2:**
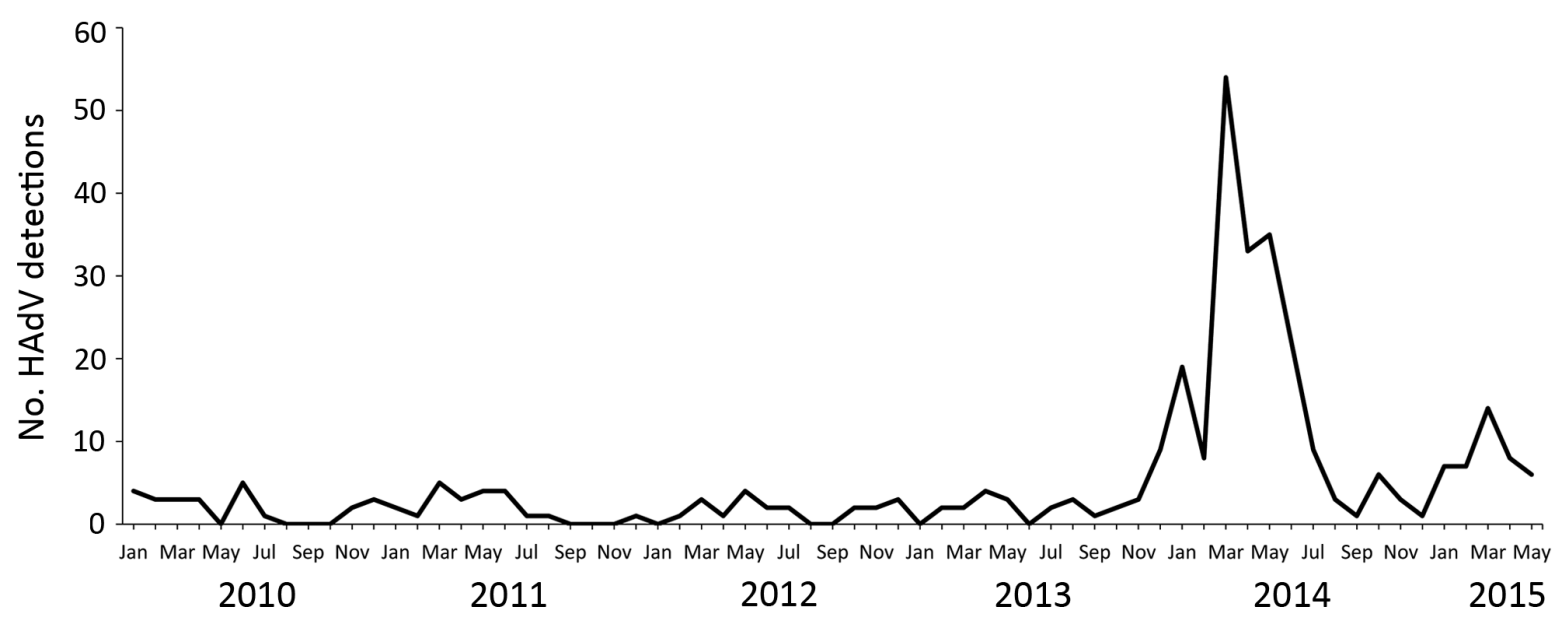
HAdV detections reported to the National Respiratory and Enteric Virus Surveillance System, Oregon, January 2010–May 2015. HAdV, human adenovirus.

Comparison to historical data for the past 3 years demonstrated an increase in HAdV reports for November 2013–April 2014. We found 2-fold and 9-fold increases in HAdV detections for the 2 large Oregon hospital systems for which historical data were available (Figure 1). HAdV detections from laboratories in Oregon reporting to the National Respiratory and Enteric Virus Surveillance System (Figure 2) increased 11-fold during the same period.

**Figure 3:**
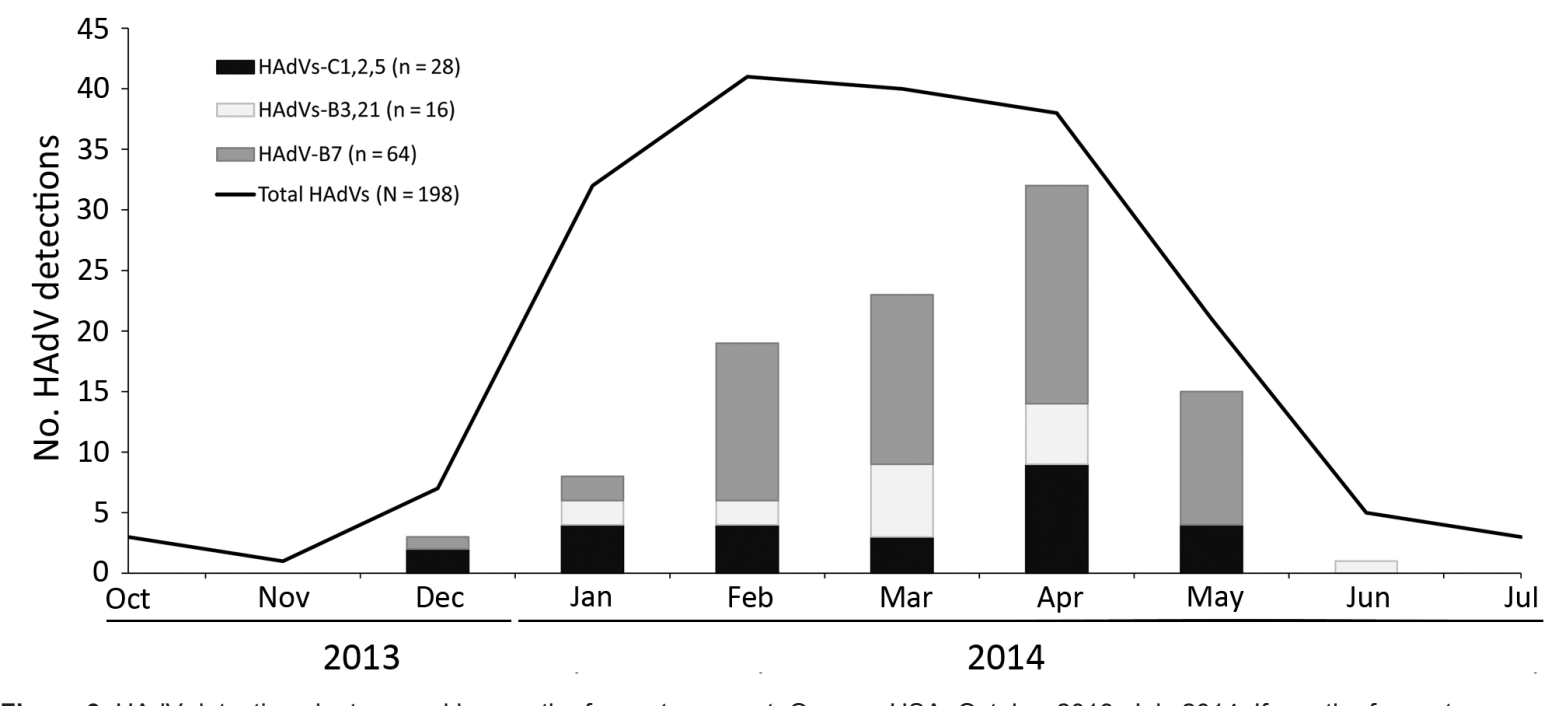
HAdV detections by type and by month of symptom onset, Oregon, USA, October 2013–July 2014. If month of symptom onset was not available, month of specimen collection was used. Total HAdVs include 109 HAdV-positive specimens that were typed (including 1 HAdV-E4 specimen) and 89 specimens that were not available for typing. HAdV, human adenovirus.

For October 9, 2013–July 7, 2014, we identified 198 patients who had an HAdV-positive respiratory specimen. Most were reported from hospital systems in Portland and surrounding counties (56%), followed by Eugene and surrounding counties (39%), Medford (4%), and 2 other locations (1%). Most (97%) cases came from 3 major hospital systems in Oregon (hospital system A, 77 [39%] cases; hospital system B, 69 [35%]; hospital system C, 46 [23%]); the remaining 6 cases came from a variety of sources around the state. Most (91%) cases occurred in residents of Oregon; the remainder occurred in residents of Washington (7%), and California (2%). Case-patients ranged in age from 3 weeks to 80 years (median 8 years); 60% were male. For most (87%) HAdV detections, symptom onset occurred or specimens were collected during January–April 2014 (Figure 3).

**Figure 4:**
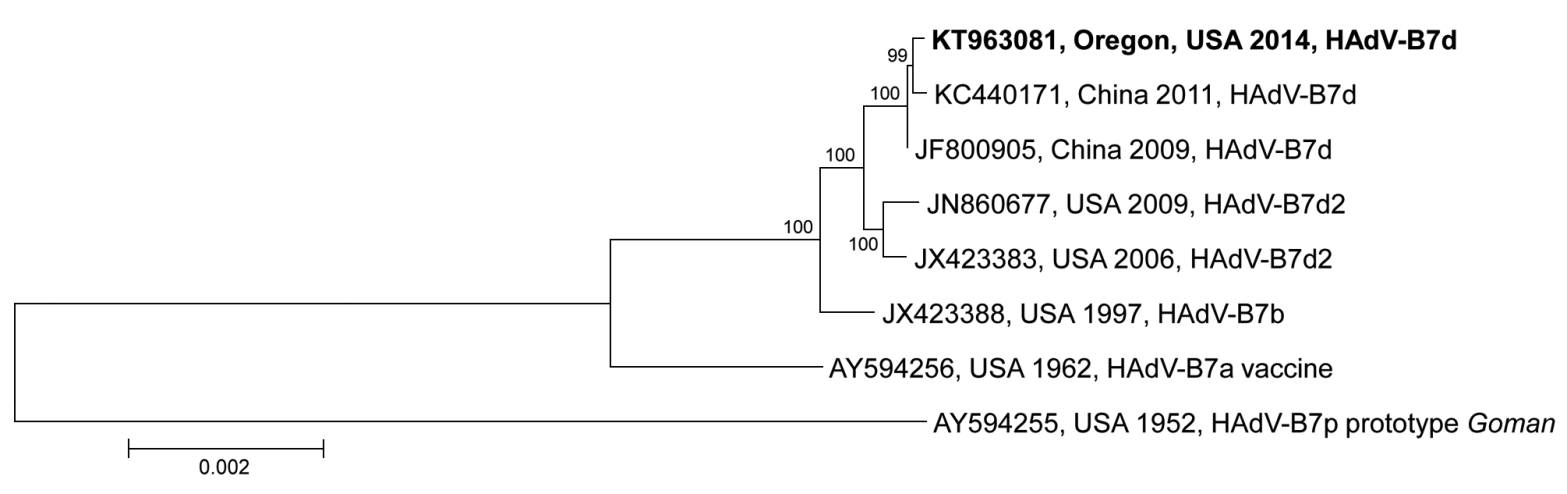
Phylogenetic analysis of human adenovirus 7 genome type d (HAdV-B7d), Oregon, USA, 2014. Genomic sequences were aligned by using ClustalW implemented in BioEdit version 7.2.5 and the neighbor-joining phylogenetic tree constructed by using MEGA7 software (*23**,**24*). Numbers at selected nodes indicate level of support using 1,000 bootstrap replicates. Sequences are identifoed by GenBank accession number, geographic location, year of sample collection, and virus genome type identified. Boldface indicates 7 identical genomic sequences identified during this study; sequences are from HAdV-B7 isolates derived from 7 different cases spanning the outbreak period. Scale bar indicates estimated number of nucleotide substitutions per site.

Specimens from 109 (55%) HAdV patients were available for HAdV typing (Table 1). We identified 7 HAdV types; HAdV-B7 was most commonly detected (64 [59%] specimens), followed by HAdV-C2 (14 [13%]), HAdV-B21 (10 [9%]), HAdV-C1 (10 [9%]), HAdV-B3 (6 [6%]), HAdV-C5 (2 [2%]), and HAdV-E4 (1 [1%]). In 2 (2%) cases, HAdV-C1 and -C2 were co-detected. The genome from 7 HAdV-B7–positive samples collected during January–May 2014 (GenBank accession no. KT963081) showed >99.9% sequence identity and gave identical in silico *BamH*I and *Bcl*I restriction profiles with a HAdV-B7d variant circulating in China in 2009 and 2011 (*22*) (Figure 4). Specimens from 89 (45%) HAdV-positive patients were not available for typing; 80% of these specimens came from hospital system A. HAdV type results were grouped by species (i.e., HAdV-C and HAdV-E), except for species B, which was grouped by type (i.e., HAdV-B7 and HAdVs-B3, -B21) to better highlight specific features of the rarely reported HAdV-B7. Most (74%) specimens tested were upper respiratory tract specimens (i.e., nasopharyngeal wash or nasopharyngeal swab samples). Case-patients with HAdV-C1, -C2, or -C5 detections were generally younger (median age 1.2 years) than those with HAdVs-B3 or -B21 (median age 24.0 years) and HAdV-B7 (median age 20.0 years) (Table 1). Case-patients with HAdV-B7 were significantly older than those with non–HAdV-B7 (Table 2).

**Table 1:** Demographic and clinical characteristics for HAdV-positive case-patients, Oregon, October 2013–July 2014*

Characteristic	Total	HAdVs-B3, -B21	HAdV-B7	HAdVs-C1, -2, -5	Not available†
Total	198 (100)	16 (8.1)	64 (32.3)	28 (14.1)	89 (45.0)
Age group, y					
Children, <18 y	118 (59.6)	8 (50.0)	28 (43.8)	27 (96.4)	54 (60.7)
<2	63 (31.8)	3 (18.8)	16 (25.0)	25 (89.3)	19 (21.4)
2–5	24 (12.1)	4 (25.0)	5 (7.8)	1 (3.6)	14 (15.7)
6–10	20 (10.1)	0	4 (6.3)	1 (3.6)	15 (16.9)
11–18	11 (5.6)	1 (6.3)	3 (4.7)	0	6 (6.7)
Adults, >18 y	80 (40.4)	8 (50.0)	36 (56.3)	1 (3.6)	35 (39.3)
19–25	16 (8.1)	0	6 (9.4)	0	10 (11.2)
26–45	19 (9.6)	2 (12.5)	10 (15.6)	0	7 (7.9)
46–65	35 (17.7)	6 (37.5)	18 (28.1)	1 (3.6)	10 (11.2)
>65	10 (5.1)	0	2 (3.1)	0	8 (9.0)
Common symptoms at presentation					
Fever	149 (75.3)	12 (75.0)	50 (78.2)	18 (64.3)	69 (77.5)
Cough	121 (61.1)	10 (62.5)	45 (70.3)	18 (64.3)	48 (53.9)
Shortness of breath	52 (26.3)	7 (43.8)	23 (35.9)	7 (25)	15 (16.9)
Nausea or vomiting	47 (23.7)	4 (25.0)	14 (21.9)	9 (32.1)	20 (22.5)
Rhinorrhea	43 (21.7)	5 (31.3)	6 (9.4)	10 (35.7)	22 (24.7)
Fatigue	27 (13.6)	2 (12.5)	15 (23.4)	3 (10.7)	7 (7.9)
Diarrhea	22 (11.1)	3 (18.8)	9 (14.1)	2 (7.1)	8 (9.0)
Sore throat	20 (10.1)	2 (12.5)	3 (4.7)	0	15 (16.9)
Myalgia	20 (10.1)	1 (6.3)	6 (9.4)	0	13 (14.6)
Wheezing	13 (6.6)	3 (18.8)	3 (4.7)	4 (14.3)	3 (3.4)
Illness severity					
Hospitalized	136 (68.7)	13 (81.3)	54 (84.4)	18 (64.3)	51 (57.3)
Admitted to ICU‡	43 (31.6)	4 (30.8)	25 (46.3)	4 (22.2)	10 (19.6)
Required mechanical ventilation‡	25 (18.4)	3 (23.1)	13 (24.1)	1 (3.6)	8 (15.7)
Died	5 (2.5)	0	2 (3.1)	0	3 (3.4)
Age, y, mean/median (IQR)§	20.6/8.0 (1.9–40)	26.8/24.0 (3–50.5)	26.8/20.0 (2.5–50.5)	3.5/1.2 (0.7–1.6)	20.6/9.0 (3–29)
Days from symptom onset to hospital admission, mean/median (IQR)‡§	4.2/3.0 (2–5)	3.6/3.0 (2–4)	4.6/3.0 (2–6)	3.9/3.0 (1–4)	4.2/ 4.0 (2–5)
Days hospitalized, mean/median (IQR)‡§	6.5/4.0 (2–8)	5.0/5.0 (3–5)	8.4/6.5 (3–9.5)	3.4/2.5 (2–4)	6.3/ 4.0 (2–6)

*Values are no. (%) except as indicated. Total HAdV also includes HAdV-E4 (1 case), which was in a nonhospitalized child (10–18 y). HAdV, human adenovirus; ICU, intensive care unit; IQR, interquartile range. †Specimens from these HAdV-positive patients were not available for typing. ‡Among hospitalized HAdV patients. §IQR = 25% and 75% quartiles.

**Table 2:** Comparison of HAdV-B7 cases with non-HAdV-B7 case-patients, Oregon, October 2013–July 2014*

Characteristic	HAdV-B7, no. (%)	Non–HAdV-B7, no. (%)†	p value
Total	64	45	
Age group, y			
Children, <18	28 (43.7)	36 (80.0)	<0.001
Adults, >18	36 (56.3)	9 (20.0)	
Illness severity			
Hospitalized	54 (84.4)	31 (68.9)	<0.05‡
Admitted to ICU§	25 (46.3)	8 (25.8)	0.06
Required mechanical ventilation§	13 (24.1)	4 (12.9)	0.26
Days hospitalized¶			
< Median, 4	14 (35.0)	19 (70.4)	<0.05
> Median, 4	26 (65.0)	8 (29.6)	

*HAdV, human adenovirus; ICU, intensive care unit †Includes only the 109 specimens that were typed. Non-HAdV-B7: HAdVs-B3, -B21, HAdVs-C1, -C2, -C5, and HAdV-E4. ‡Not significant after adjustment for age. §Among hospitalized case-patients. ¶A total of 40 HAdV-B7 case-patients and 27 non–HAdV-B7 case-patients had both hospital admission and discharge dates.

For all case-patients, the most common symptoms were fever (75%), cough (61%), shortness of breath (26%), nausea or vomiting (24%), and rhinorrhea (22%) (Table 1). Pneumonia was reported for 32% of the 198 HAdV patients (41% of those with HAdV-B7, 31% of those with HAdVs-B3 or -B21, 21% of those with HAdVs-C1, -C2, or -C5). A total of 136 (69%) persons were hospitalized (Table 1), and 62 (31%) were outpatients seen in the emergency department or in outpatient clinics. HAdV-B patients were more often hospitalized (HAdVs-B3 or -B21: 81%; HAdV-B7: 84%) than HAdVs-C1, -C2, or -C5 (64%) patients (Table 1). Among hospitalized case-patients, 32% were admitted to the ICU, and 18% required mechanical ventilation (Table 1); 46% of HAdV-B7 case-patients were admitted to the ICU (Table 1). Median length of hospital stay for HAdV-B7 case-patients was longer (6.5 days; interquartile range [IQR] 3–9.5 days) than for HAdVs-B3 or -B21 case-patients (5.0 days; IQR 3–5 days) and HAdVs-C1, -C2, or -C5 case-patients (2.5 days; IQR 2–4 days). However, when we compared HAdV-B7 with non–HAdV-B7 case-patients, only length of hospitalization remained statistically significant after adjustment for age; HAdV-B7 case-patients were significantly older (adults) and hospitalized longer than the median (4 days) than non–HAdV-B7 case-patients (p<0.05) (Table 2). Five (2.5%) case-patients died; for 2, specimens were available for typing, and both were HAdV-B7.

## Discussion

We describe a community outbreak of HAdV in Oregon where HAdV-B7 was identified as the predominant HAdV type. HAdV-B7 was more often detected in adults than children and was associated with more severe disease than other HAdV types. In persons <18 years of age, 7 HAdV types were detected, whereas in most persons >18 years of age, HAdVs-B3, -B7, or -B21 were detected. These findings are consistent with other studies showing that HAdVs-C1, -C2, and -C5 are most frequently detected in young children (*17*,*25*). Many studies have demonstrated severe illness associated with HAdV-B7 in children or in military recruits, but few have documented such a widespread community outbreak in which many adults experience severe respiratory illness (*5*,*18*,*26*,*27*). However, a high percentage of adults with severe respiratory disease has been reported in other community outbreaks of related HAdV-B viruses (*6*,*13*,*28*,*29*).

Compared with non–HAdV-B7 case-patients, HAdV-B7 case-patients were more frequently admitted to the ICU and mechanically ventilated, were significantly older, and had significantly longer hospital stays. Our findings of severe infection with HAdV-B7 requiring ICU admission and extended hospitalization is consistent with other reports linking HAdV-B7 with more severe acute respiratory disease (*30*–*32*). HAdV also was detected in 5 fatal cases, 2 of which were identified as HAdV-B7. Although the contribution of HAdV to a patients’ death is unclear, similar reports of severe outcomes associated with HAdV infection have been documented (*18*,*26*,*30*).

Further molecular analysis showed that genome sequences of 7 Oregon HAdV-B7 isolates were identical to each other and nearly identical to strains circulating in China in 2009 and 2011. Predicted restriction enzyme profiles have not previously identified the genome of this virus (HAdV-B7d) in the United States (A. Kajon, pers. comm.) until recently (*33*). HAdV-B7d was first isolated in China in 1980, where it became the predominant circulating genome type at least through 1990 (*22*) but then disappeared until reemerging 21 years later (*15*). Severe respiratory disease and higher case-fatality rates have been associated with HAdV-B7, especially HAdV-B7d. Enhanced virus fitness, low herd immunity to HAdV-B7, or both might have contributed to the large community outbreak in Oregon.

The US military used an effective live oral vaccine for HAdV-E4 and HAdV-B7 from 1971 until 1999, and the vaccine was reintroduced in 2011 (*34*,*35*). During 1999–2011, when the vaccine was unavailable, vaccine-preventable HAdV infections increased substantially in US military personnel (*36*). Subsequent reintroduction of the vaccine in October 2011 resulted in a significant decrease in these HAdV infections, indicating HAdV vaccination is an effective prevention measure for HAdV infection in this setting (*35*). However, currently no HAdV vaccine is available for use in the general public.

Our study has several limitations. The HAdV cases described in this case series do not represent a population-based sample and might not represent all HAdV cases in Oregon during this period. Persons with milder illness were not sought out at participating facilities because case finding was primarily conducted in hospitals and because the Health Alert Network notice requested providers to consider HAdV in patients with severe pneumonia. Because of the timing of the investigation, almost half of the HAdV-positive specimens were not available for typing (many were cases diagnosed earlier in the study period), and 80% of these specimens came from 1 of the 3 major hospital systems that participated in the investigation. The molecular methods used for typing HAdVs in this study targeted the hexon hypervariable regions that have been shown to correlate closely with virus serotype. These methods do not provide genomic detail and might miss recombination events in other regions of the virus genome. Moreover, full-genome sequencing was performed on only 7 HAdV-B7 isolates, and although they were chosen from different time points in the outbreak, we cannot conclude that all HAdV-B7 detections were HAdV-B7d. Finally, detection of HAdV in a respiratory specimen, especially HAdV-C, does not necessarily imply causation because asymptomatic HAdV shedding can occur. HAdV-C detections in infants and young children with mild respiratory infections and the virus might persist in the adenoids and tonsils and be shed for prolonged periods after symptoms resolve (*37*–*39*). The clinical significance of HAdV latency in tonsil and adenoid tissue is unclear.

HAdV type surveillance is an important tool for monitoring changes in predominant types and genome types. Shifts in HAdV types might be associated with more severe disease not only in vulnerable populations, such as children, elderly persons, and immunocompromised persons, but might also cause community outbreaks of severe respiratory disease in adults, as occurred in Oregon. Healthcare providers should consider HAdV in their differential diagnosis for patients with pneumonia and acute respiratory infection. Testing for HAdV using respiratory panel PCR assays and HAdV typing has been increasing nationwide. In response, CDC recently launched a voluntary and passive surveillance system to collect HAdV typing data from laboratories called the National Adenovirus Type Reporting System. The main objectives of this system are to better define circulation patterns of HAdV types and better monitor HAdV outbreaks in the United States.

HAdV-B7 might be reemerging in the United States and might be associated with increased numbers of severe respiratory infections. Tracking the emergence of HAdV types in the United States will lead to early identification of new types and potential variants of known types. Our results demonstrate how HAdV surveillance might help explain clusters and sporadic cases of severe illness possibly related to changes in HAdV species.
